# Multicultural Neurolinguistics: A Neuroscientific Perceptive of Cross-Cultural Differences in Translation

**DOI:** 10.3389/fpsyg.2022.939517

**Published:** 2022-07-12

**Authors:** Wei Huang, George Kwame Agbanyo

**Affiliations:** ^1^Department of International Cooperation and Exchange, Honghe University, Mengzi, China; ^2^School of Business, Honghe University, Mengzi, China

**Keywords:** cultural diversity, cross-cultural differences, psycholinguistics, neurolinguistics, translation

## Abstract

Psycholinguistics and neurolinguistics have been seldom used in investigating the cultural component of language. In this study, we suggest a scientific methodology to study neurocognitive mechanisms induced by the interaction between multi-linguistics and cross-culture differences, especially during translation between a source language (SL) and a target language (TL). Using a contest of tonal languages (Chinese) and atonal language (English) multilingual exchange, we opine that translation theories as numerous and efficacious as they are, lack the competence to bring absolute clarity into the complex cross-cultural dimension of languages when it comes to accuracy in translation. Echoing this, this study attempts to apply neuroscience in blending cross-cultural diversity and neurolinguistics as a one-in-all translation approach to “multicultural neurolinguistics” between an SL and a given TL. The linguistic examination of this study proves that “multicultural neurolinguistics” will provide a unique framework for all translation barriers, and establish a cross-cultural and multilingual network depending on the particular circumstance. This research contributes to the linguistic literature by bringing a “multicultural neurolinguistics” resolution to the cultural diversity question in translation.

## Introduction

Referred to as the operation of substituting from language into another, translation constitutes of reproducing messages in a target language (TL) closely and naturally, commensurate with the one in a source language (SL), in terms of meaning and style (Nida, 1993). According to this position, translation affects the structure, meaning, and style, while still holding together the linguistics, semantics, and stylistics of the TL. So the expectation that if a person is fluent in two or more languages, he or she should be able to translate easily, is questionable. Actually, accurate text comprehension and rendering are more likely to be a matter of cultural variety than of language variations. According to Nida (1993), cultural differences provide the most important obstacles for translators and have resulted in the most widespread misconceptions among readers. Echoing this, to a large extent, translation has a direct effect of structure, meaning, and style of the TL. Moreover, translating requires the ability to master several languages, linguistic aspects and writing style, as well as the ability to integrate those various masteries. These requirements are considered as foundational for communicative translation to help one to become more conscious of the limits of both the SL and TL. Language in the context of translation is considered as a communication instrument in which a piece of linguistic structure may reflect a variety of meanings depending on context, circumstance, involvement, goal, and a variety of cultural elements. Because language is multifunctional, it cannot be isolated from its contents and paralinguistic aspects. Language is the primary substance of human symbolic action, and it is a complex substance, and even an ecosystem of sociolinguistic systems (Ellis et al., 2015).

Translation is the first route of communication par excellence between nations meeting for the first time (Pérez-González, 2019). Consequently it is the principal avenue for transferring believes, thoughts i.e., Culture. Exploring cultural differences and their influence on translation decisions has recently become a primary concern for linguistic scholars and translators. While translation is mostly seen as a form of cross-cultural communication, cultural differences provide significant difficulties to translation. Echoing this, translation can be seen as the only linguistic channel for overcoming cultural incorrespondencies. Following a short research of cultural variations in translation, this perspective article focuses on scientific “multicultural neurolinguistic” approaches for dealing with cultural differences and the variables that may influence the choice of translating theories. By proposing a “multicultural neurolinguistic” solution to cross-cultural interference in translation approaches, this work addresses a significant vacuum in the multi-linguistic and translation literature. This research believes that a “multicultural neurolinguistic” analysis of culture and linguistic interaction in the brain will give a comprehensive approach to multilingual knowledge transfers in translation.

## Overview of Theoretical Foundation in Translation

### Translation and Cross-Cultural Identity

Obviously, defining translation as the act of transferring or copying from one language to another is inadequate since it neglects the peculiarity of each language (Nida, 1993). In addition to the lexical, semantics, and grammatical structures, translation is a craft that is used to faithfully replace written information in one language with the same information in another language. However, even with all these dispositions, translation is not always able to deliver the message faithfully, because of words, sentences, and structure differences between SL and TL. Because the main objective of translation is the flip of words from one language to the other, but rather to relate create a flow of knowledge between cultures without distorting the content and context of any particular situation. Practically, translation is seen more like a cultural agent since it plays the role of mediator reconciling cultures (Pérez-González, 2019). To some extent, the vocation of translation is to transcribe cultural references by adapting to detours of the both SL and TL, especially in the case of no direct words and expressions in the TL, as the old Latin legend puts it “Traduttori traditori” (translators traitors).

Besides structural elements, one of the underlying obstacles to translation is the cultural background of each language. Translation, as seen from this perspective, is more than just shifting from one language to another; it is the interpretation of a source linguistic version into another linguistic target. As such, translation, unlike grammatical and structural frameworks, represents the problems of cross-cultural variety and language heritage. This is particularly risky when interpreting official papers in a corporate or legal setting. There are ~7,000 languages in the world, each with its origin, roots, and structure, while one language could also have derivatives with variations of different degrees (Nida, 1993).

### Linguistic Differences and Translation Techniques

Traditionally, since translation is largely an interlingual communication, the primary issues of contention amongst translation theories are essentially linguistic variations in expression. One of the earliest references, Vinay and Darbelnet (1958), published “Stylistique comparée du français et de l'anglais: Méthode de traduction; Comparative Stylistics of French and English: A Methodology for Translation” which quickly became a translation manual among scholars in the West. Coincidentally in the same year, Loh (1958) textbook “英汉翻译的理论与技巧” “Translation: Its Principles and Techniques” was very instrumental and gained popularity. Both master pieces emphasize taxonomies to describe and analyze linguistic changes in translation and today their model “shift approach to translation” is widely adopted among scholars. Table 1 below is the summary of the combination of both authors.

**Table 1:** Model of translation principles and techniques.

**General translation methods**	
**Direct, literal translation (直译)**	**Oblique, free translation (意译)**
**Translating words and expressions**	
Part one	Ways for translating nouns denoting things of foreign origin
	Borrowing, Transliteration (音译): e.g., chocolate 巧克力
	Semantic translation (语义翻译): e.g., airplane 飞机
	Combination of transliteration and semantic translation (音译兼语义翻译): e.g., utopia 乌托邦
	transliteration plus semantic translation (音译加语义翻译): e.g., beer 啤酒
	symbolic translation with a semantic explanation (符形翻译加语义注释): e.g., cross †字架
	Calque, coinage of new characters(造新词语): e.g., oxygen 氧
Part two	Change of parts of speech in the translation process: The translator must master the SL and TL
	Relatives, demonstratives, indefinites, interrogatives, articles, verbs, modifiers, numerals, connectives
**Comparative study of the languages**	
Part three	Major differences between Chinese—English: the translator must master the SL and TL
	Word-formation
	Morphology
	Syntax
Part four	
Principles/techniques (In order to conform to the TL standards)	Detailed items: the translator must master the SL and TL
Omission (used to solve apparent paradox, focus on the necessary, indispensable, remove the useless and superfluous)	Subjects, personal pronouns, articles, modifiers, e.g., *She covered her face with her hand as if to protect her eyes* 她用手蒙脸，好像去保护眼睛 *(She used … hand cover … face, as if to protect … eyes)*
Amplification (add subject, pronouns, articles, connectives, and prepositions)	Syntactic items: subjects, pronouns, e.g., …不了解这一点，就不能得到起码的知识. …* Unless we grasp this point we shall never be able to acquire even elementary knowledge (Not grasping this point never be able to acquire even elementary knowledge)*
	Semantic items: supplementary words, summarizing words, illustrating words, connecting words e.g., *She is sitting in a Ford* 她坐在福特汽车里. *(She sits in a Ford car)*
Repetition (for clarity, for emphasis and for vividness)	Subjects (nouns), verbs, objects, adjectives, e.g., Paul had it all written out neatly 保罗把它写得清清楚楚 (Paul had it all written out neatly and clearly)
Transposition, conversion (substituting SL words with TL words identical in meaning but different part of speech)	Nouns → verbs, adjectives, adverbs and vice versa English adjectives → Chinese adverbs English genitives/possessives → Chinese nominal/objective nouns English adjectives/participles → Chinese nouns English prepositions → Chinese nouns English adjective clauses → Chinese adverbial elements e.g., *A lesson to all* 给大家一个教训. *(Give everybody a lesson)*
Modulation, inversion	Positions of subject and object, complement, adjective modifiers/clauses, adverbials of manner/place/time e.g., *I don't even know his name* 我连他的名字都不知道 *(I even his name (do) not know)*
Modulation, negation	In ways of thinking, word-formation, idioms *e.g., Is he right?” “I don't think he is right”* “他对不对？” “我想他不对” *[“He (is) right or not right?” “I think he (is) not right”]*

*Data extracted from: Vinay and Darbelnet (1958) and Loh (1958)*.

Vinay and Darbenet's work was a comparative stylistic analysis based on a French-English, French-German and English-Spanish translation. They identified two major methods of translation: literal and free translation, and mostly use the term “procedures” to refer to translation (Table 1 part1). Even though, Loh also identified the literal translation (直译) and free translation (意译) methods, he used the term “ways” (方法) and “principles and techniques” (原则与技巧), instead of “procedures,” to argue that translation being a bilingual art could only be done by an individual who masters both SL and TL. His model is a very detailed lexical and structural analysis (Table 1 part 1, 2, 3, 4).

### Cross-Cultural Differences and Translation Challenges

Each translation technique has advantages and disadvantages, depending on the situation and cultural background or an individual's preferences. Consequently, several elements should be examined before deciding on the approach or procedures to utilize. Nida (1993) identified four criteria that are likely to impact the selection of cultural technique. They are the aim of the translation, the kind of text, the author's intention, and the audience (Ellis et al., 2015).

**Figure 1:**
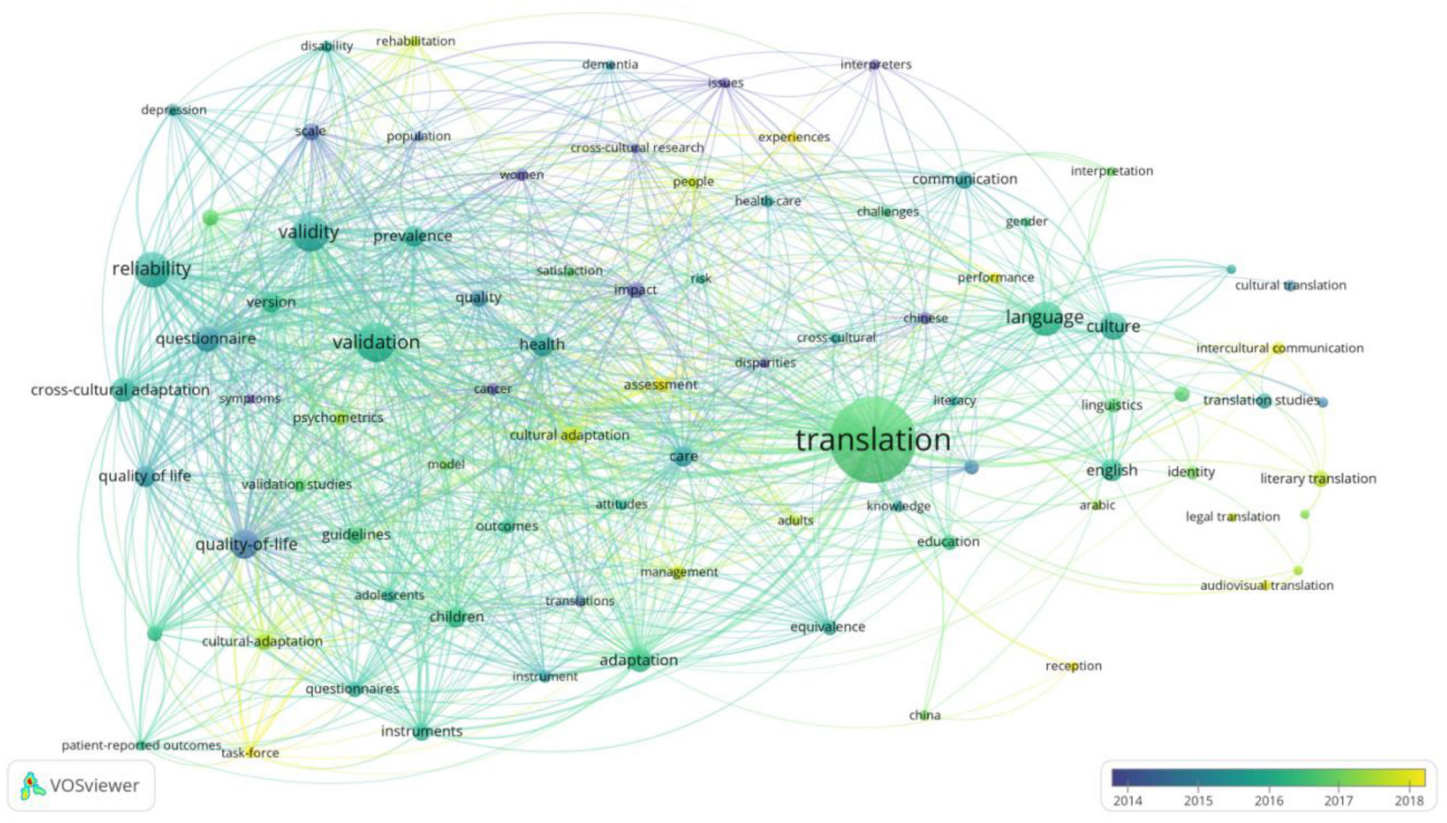
Bibliometric analysis keywords “Neuroliguitic*” “Translat*” “Cultur*.”

Language is the lifeblood of culture, as culture is the path along which language emerges, is acquired and grows (Hofstede, 1980). Language is no exception to the fact that the genesis and evolution of all components of culture are inextricably linked. A comprehensive examination of word meanings and how they evolve demonstrates how culture influences language genesis and growth. As clearly seen in the bibliometric analysis result in Figure 1, language is a result of cultural evolutionde (de Boer and Thompson, 2018). This distinguishing element of Western civilization is fully exemplified in Indo-European languages, particularly English, which is largely a synthetic and analytic language distinguished by grammar, hypostasis in syntax and flexible word construction. In contrast, the Chinese of the rich East Asian continent is characterized by rich parataxis and simplistic grammar (Chin et al., 2021a). Chinese, as an analytic language, mirrors the psychological characteristics of the Chinese people (Shweder, 1991). From the fundamental linguistic differences, it could be concluded that language, as an institutional component of culture, is intimately tied to and heavily impacted by other aspects of culture. The disparities between the Chinese and English languages are mostly due to the cognitive and psychological differences between the two cultures. As a result, the majority of translation misunderstanding happens because of limited knowledge of the cultural differences and similarities between a SL and a TL. Therefore, translation cannot be left entirely to artificial intelligence.

To substantiate the implication of culture in the evolution of language, a bibliometric analysis was conducted to explore the dominant clusters and possible linkages established in literature through the years (Chin et al., 2021b). Because of its cross-disciplinary mapping and mega-data handling abilities, the bibliometric was employed to bring clarity and backup the aspect of our argument concerning the important role of culture in the emergence, acquisition and growth of language, however, literature has failed to give much consideration to culture in linguistic translation as seen clearly in the Figure 1 below. For the bibliometric analysis we used different combinations of the keywords; “Neuroliguitic^*^, Translation^*^, Cultur^*^,” through the Web of Science (WoS) search to collect the data. Because the objective of this analysis is to demonstrate the research trend of this subject, we did not apply any restriction for the data collection and we obtained 2, 717 documents ever published according to WoS. When the data was processed through Vosviewer, and number of occurrence was adjusted to 15, 108 documents remained for the network visualization in the Figure 1 below.

The bibliometric results as seen above shows “Translation” as the most significant cluster, with considerable occurrence in literature only after 2016. Translation has the closest connections with “Language” and “Culture” with the latter two having a weaker cluster. Other keywords like “Cross-culture” “communication” “linguistics” also occurred in the network but very weak cluster and a distant connection with translation. In support of the argument of this perspective article, despite the significance of cross-culture identity in the emergence, acquisition and growth of language, literature records a dearth of established authority research on the subject (Chin et al., 2021b). In regard with the intricacy between culture and language, the result reflects the lack of research to elucidate how the connection between the two functions. To address the question, we took the discussion further to conduct a neurolinguistic investigation on the implication of cultural on language and translation. We attempted a bibliometric analysis to identify the trend in literature on the subject and unsurprisingly the results were nothing to write home about. Keywords such as Neurolinguistics, culture and translation were used in various ways but they all show a remarkable research gap on the subject. First, “Neurolinguist^*^” “Cultur^*^” “Translat^*^” were processed through the WoS search tool with no restriction, the result were 6 published documents (five articles, one meeting) from 1979 to 2021. The next combination “Neurolinguist^*^” “Translat^*^” showed 40 publications (34 articles, 6 books) between 2017 and 2021. Another keyword combination “Neurolinguist^*^” “Cultur^*^” gives 51 published documents (45 articles, 4 books, 1 clinical trial, and 1 report) between 1919 and 2021. With these results, this paper tries to deepen the discussion by a neuroscientific investigation of the linguistic process in the brain and further analyse how the brain captures the implication of culture on the linguistic process in order to establish a neurolinguitic (scientific) instrument aid for translation purposes.

## Psycholinguistics and Neurolinguistics Across Language Diversities

### Psycholinguistic and Neurolinguistics Interpretation of Languages

The study of the interrelationship between linguistic and psychological elements is known as psycholinguistics, which is concerned with how language is processed and represented in the mind; that is, the psychological variables that allow people to learn, utilize, understand, and generate language. Psycholinguistics is an interdisciplinary field involving psychology, cognitive science and linguistics, and is more and more overlapping with neurolinguistics due to recent scientific discoveries. Neurolinguistics on the other hand looks at where language information is processed, how language processing evolves over time, how brain structures connect to language acquisition and learning, and how neurophysiology may help with speech and language disorders. Much of the early work in neurolinguistics, such as Broca's and Wernicke's, studied the locations of particular language “stimuli” throughout the brain. Meanwhile, further, advances in neurolinguistics have explained the stages of language development in correlation with the growth process of the human brain by proving how Infants go through similar and specific stages of speaking. Further technical advances such as Hemodynamic (e.g., PET and fMRI) use blood flow observations in the brain to determine the relation between parts of the brain and languages (Wong et al., 2004; Bender, 2020). Other neuroscientific techniques such as EEG, MEG and TMS use electric and magnetic field activities to detect brain activity at a millisecond degree of stimuli, very instrumental for language comprehension and production (Cheng et al., 2022).

For a crosslinguistic examination, neural differences in language diversity are explored based on scripts, orthography, and tonality. Scripts which refer to the system of writing a language have two categories such as the alphabets and logographs and are closely related to the orthography which refers to the rules of writing each language, thus transparent and non-transparent orthography (Bender, 2020). Then, tonality which concerns the pitch of speech indicates identity, affection, intonation, phonemic stress, and word meaning (Wong et al., 2004). Consequently, the neural mechanism correlates directly with these cross-linguistic variations. Bolger et al. (2005) and Gandour (2005) recently reviewed 43 research works on different languages to find significant cross-language different activations in the left middle frontal gyrus, temporoparietal region, and right fusiform cortex. Findings reveal greater activation of the right occipitotemporal region for logographic system processing than alphabetic processing (Bolger et al., 2005) Obviously, the logographic system (Chinese characters), with intriguing strokes, involves more visuospatial neural processing than the linear combination letters of the alphabetic system. For orthography, research reveals that transparent orthography (Italian) induced more reactions in the left posterior superior temporal region whereas quasi-transparent orthography (English) caused more activation in the left posterior inferior temporal gyrus. However, for Chinese (non-transparent orthography), the inferior dorsal parietal lobule is more activated, perhaps because it involves visuospatial analysis. Concerning lexical tones, the research compared tonal languages (Chinese and Thai) and atonal languages (English) to find that tonal language showed more left-lateralized activations in the front-temporal regions than atonal languages (Gandour et al., 2003). This reveals the central role of the left hemisphere in expressing lexical tones instead of the right hemisphere. At the same time, findings reveal that neural patterns for the first and second languages, especially for Chinese and English are not more distinct. Wang et al. (2003); Han and Northoff (2008) recently found a kind of enrichment plasticity in which cortical regions are enlarged and/or recruited to perform new language functions that are comparable to their original language idiosyncrasies.

### Neurolinguistics Interpretation of Cross-Cultural Complexities

Neurolinguistics provides an understanding of the language mechanism in the brain, but the identification of culture as a sub-set of language has not been well-documented in the literature. Researchers like Bolger et al. (2005) have begun to investigate in detail the effect of language as a cultural medium and the culture multidirectional relation on the brain process (Han and Northoff, 2008). The element of culture is fundamentally explained by two neurobiological principles: Neural plasticity which explains the imprint of culture on the brain, and neural specialization which sustains cross-cultural differences in the brain (Ellis et al., 2015; Chin et al., 2021a).

As previously stated linguistic elements such as scripts, orthographies, and tonality influence the neurological basis of language acquisition through neural specialization, which is an extension of the classic case of neural specialization in phonetic processing. Neuronal plasticity, which allows for accommodation, works in concert with neural specialization. Indeed, there is sufficient evidence that language acquisition may alter brain functioning and anatomy as a result of neural plasticity. Phonetic training, for example, may cause functional restructuring, such as the expansion (plasticity) of some regions of the brain (Wang et al., 2003; McBride-Chang et al., 2004). Language acquisition has also been shown to cause persistent changes in brain anatomy (Wu et al., 1998). When compared to monolinguals, bilinguals had higher gray matter density in the left inferior parietal area (Mechelli et al., 2004). This dynamic process of adaptation and assimilation, or plasticity and specialization, is expected to occur across all elements of culture–brain relationships and throughout the course of a person's life (de la Fuente et al., 2014). So far, researchers have only discovered a few examples, such as phonetic and visual word processing.

To a large extent, the multilingual mechanism of cross-cultural processes in the brain is beyond neurolinguistic demonstration. Therefore, this research work perceives a more holistic interdisciplinary field such as “Multicultural neurolinguistics” as a strategic instrument best fit for a scientific approach for translation studies. From the perspective of the multicultural neurolinguistic field, the neural reaction map of cross-cultural and language diversities will be a monumental paradigm shift for the translation discipline. Echoing this, multicultural neurolinguistics will be strategic to provide a scientific translation chart of languages across cultural identifications (de Boer and Thompson, 2018; Cheng et al., 2022). Evidently, multicultural neurolinguistic studies will disclose, to a deeper extent, the within-group and the within-individual cognitive diversities. A comprehensive application of multicultural neurolinguistic measures in the decision-making process of translation techniques is very instrumental for bridging the cultural difference complexity. This paper perceives multicultural neurolinguistics to establish, on one part, cultural networks for spoken and written languages and on another specific language activated neural regions, and then connect neural bases of different scripts, orthographies, and tonalities. It also creates room for a neural basis of second language and the role of native language experience in a second language acquisition. This will be a general neuroscientific road map to integrate culture and linguistic neural bases and then discuss future research areas concerning emerging linguistic development (Nakada et al., 2001).

## Discussing

The development of language systems (phonology, morphology, syntax, and script) in the human brain has been greatly aided by the discovery of neuroscience. Furthermore, new research in neurolinguistics have been focused with how culture—a fundamental component of language conception—interacts with the cognitive process of language (de la Fuente et al., 2014). Neurolinguistics is still in its early stages of investigating the distinctions in the brain foundation of various languages. This discussion introduces a novel platform “multicultural neurolinguistics,” which elucidates the cross-cultural component of language cognition, and how it can practically been used in translation. The next paragraphs argue for the theoretical and practical use of “multicultural neurolinguistics.”

### Theoretical Implication

As a complex behavior, language requires several senses and motor abilities, as well as their coordination, whereas different languages are characterized by a variety of aspects, such as phonology, morphology, syntax, semantics, and scripts (Quan et al., 2021). Researchers have classified the more than 6,000 human languages into main language groups based on these distinctions; e.g., Niger-Congo, Austronesian, Sino-Tibetan, Indo-European, and Afro-Asiatic, with each containing hundreds of languages. These cross-cultural changes in scripts, spelling, and tone may have a major impact on language processing brain systems (Wu et al., 1998; Wong et al., 2004).

According to recent neurolinguistics discoveries, visual arrangement differs greatly across various writing systems or scripts. One example is the contrast between alphabetic and logographic systems. Logographic systems (Chinese characters) may need more visual examination than alphabetic texts (English alphabets). Visual analysis might be bilateral or right-hemisphere dominating (Grill-Spector, 2001). Existing neuroimaging studies on Chinese processing have demonstrated bilateral or even right-dominated activity in the occipital and posterior occipitotemporal regions, supporting this theory (Tan et al., 2000; Siok et al., 2004). Also languages rely on different phonological access methods depending on transparent and non-transparent orthography. These various phonological access methods entail diverse brain processes. Reading English engaged the posterior superior temporal gyrus and adjacent supermarginal cortex, but reading Chinese activated the dorsal portion of the inferior parietal lobule, perhaps due to its involvement in visuospatial interpretation of Chinese characters (Tan et al., 2005). According to recent neuroimaging findings, lexical tone activates the left inferior frontal areas and the temporal regions (Wang et al., 2003; McBride-Chang et al., 2004). Moreover, Cross-linguistic studies even provide evidence for the influence of linguistic characteristics or language experience on lexical tone processing. Several studies have compared neural mechanisms of tone processing in speakers of a tonal language (e.g., Chinese and Thai) with those of an atonal language (e.g., English) and discovered that speakers of a tonal language had more left-lateralized activations in the frontotemporal regions than speakers of an atonal language (Gandour et al., 2003; Wong et al., 2004; Gandour, 2005). There is evidence that the left hemisphere is more successful than the right hemisphere in acquiring lexical tones (Wong et al., 2007).

To process from research works on cultural variations in the brain based on voice processing and script of languages, this study proposes cross-cultural differences in the neurological bases of other elements of language processing and translation. Verbs, for example, are frequently represented in the frontal area in studies of English and other Indo-European language users, while nouns are represented in the posterior regions (Petersen et al., 1989). However, nouns and verbs in Chinese engage a diverse set of overlapping brain regions in dispersed networks in both the left and right hemispheres (Li et al., 2004). The explanation for this cross-cultural variation is most likely because word classification into distinct grammatical classes in Chinese is less clear-cut than in English. Many individual words in Chinese are difficult to recognize as nouns or verbs, owing to a lack of inflectional morphology in Chinese. Most words serve numerous grammatical functions, resulting in a plethora of class-ambiguous terms that may be employed as nouns or verbs. To address the cross-cultural intricacies in the language mechanism “Multicultural neurolinguistics” is the perspective presented by this paper as the research framework equipped to fully comprehend cross-linguistic differences in the neurological substrates of semantic processing.

### Practical Implication

As mentioned earlier, cultural neurolinguistics is only at the beginning stage of development. Thus far, most research has focused only on the effects of cultural features on the language brain. This field needs to address both classic and new questions such as how the interaction between the features of languages and the brain anatomy and function affect the neural basis of different languages, how social factors (e.g., social economic status, education, vocabulary and knowledge explosion, technology use, communication style, economic, cultural orientation, etc.) that shape the language use and experiences would shape the brain (Raizada et al., 2008; Agbanyo and Wang, 2022), and how language and the brain coevolve to create the diversity in languages and the diversity in neural bases of languages (Han and Northoff, 2008; Ellis et al., 2015). There are three distinct ways, occurring at different stage of development, in which cultural factors might help mold the human brain. First, the ecological surroundings associated with a certain culture may selectively tune appropriate neuronal connections. Second, cultural factors in early child learning differentially and dynamically alter brain development. Third, life-long adaptability allows the adult brain to continuously adapt to new situations (Raizada et al., 2008). Moreover, even though, preexisting brain circuitry places structural constraints on the brain-cognition mapping, brain plasticity allows flexibility in the specifics of the mapping. Language learning can change brain functions and even anatomy due to neural plasticity. Language learning can also result in permanent changes in brain structure (Quan et al., 2021). Bilinguals have been found to show increased gray matter density in the left inferior parietal region as compared to monolinguals (McBride-Chang et al., 2004; Mechelli et al., 2004). This dynamic process of accommodation and assimilation or plasticity and specialization is likely to occur across all aspects of culture–brain connections, and across the life span (de Boer and Thompson, 2018).

Cross-cultural differences in neural bases of speaking and reading reveal cross-cultural differences in neural bases of other aspects of language processing. Studies on English and other Indo-European languages have confirmed that verbs are represented in the frontal region, whereas nouns are represented in the posterior regions (Petersen et al., 1989; Siok et al., 2004). Nouns and verbs in Chinese, however, activate a wide range of overlapping brain areas in distributed networks, in both the left and the right hemispheres (Li et al., 2004). The reason for this cross-cultural difference is probably that categorization of words into different grammatical classes is less clear-cut in Chinese than in English. In Chinese most words play multiple grammatical roles, resulting in an abundance of words that can be used as either nouns or verbs. Much more research is needed to understand crosslinguistic variations in the neural bases of semantic processing, more particularly for translation purposes. Thus far, this research has focused on comparisons of neural bases of different languages and different cultures and how that can help in translation. Cultural encounters lead to exposure to and acquisition of second languages. Neural bases of second language and especially in translation where the role of native language in second-language acquisition are important to establish relatively more accurate translation systems. Multicultural neurolinguistics could be the cross-disciplinary framework to discuss questions on how cross-culture, neurolinguistics interact in translation designs. So far, no neuroimaging study of such a design has been conducted.

## Research Limitation and Future Direction

The intimate relations between language and culture have long been the hot point of discussions by scholars and experts in many fields and disciplines (Siok et al., 2004), especially during recent years of the global village, when intercultural exchange and translation have become inevitable (Petersen et al., 1989). Any language is considered as a symbol representing a particular cultural identity (Li et al., 2004); therefore, translation from an SL to a TL is forcibly transferring the culture it represents and embodies (de Boer and Thompson, 2018). Meanwhile, with recent neurolinguistic discoveries, the neural metabolism of the language process and the corresponding part of the brain is largely mapped out (McBride-Chang et al., 2004; Wong et al., 2004). However, the implication of cultural diversity on the cognitive mechanism has not been adequately explored, let alone in relation to language (Raizada et al., 2008). To establish a firm foundation for cross-cultural and multilingual patterns, this study puts forwards the intriguing proposition of the “multicultural neurolinguistic” framework as a strategic scientific field adequate to accommodate the intricacies of the neural mechanism concerning cross-cultural diversity in the multilingual context. As a novel research field “multicultural neurolinguistic” is perceived as a strategic instrument to surmount all translation barriers in the context of cross-cultural and language diversities. Still further studies could employ such multidisciplinary framework as a strategic scientific instrument to bridge cross-cultural barriers in international trade. International economic transactions uncontestably promise a better market within a “multicultural neuroeconomic” framework.

## Data Availability Statement

The original contributions presented in the study are included in the article/supplementary material, further inquiries can be directed to the corresponding author/s.

## Author Contributions

GA designed and conducted the research and also wrote the first draft of the manuscript. WH searched and analyzed the literature and wrote the main part of the manuscript. GA and WH contributed to the article and approved the submitted version.

## Conflict of Interest

The authors declare that the research was conducted in the absence of any commercial or financial relationships that could be construed as a potential conflict of interest.

## Publisher's Note

All claims expressed in this article are solely those of the authors and do not necessarily represent those of their affiliated organizations, or those of the publisher, the editors and the reviewers. Any product that may be evaluated in this article, or claim that may be made by its manufacturer, is not guaranteed or endorsed by the publisher.
